# A84 COMPETENCY BASED MEDICAL EDUCATION: O-SCORE CHARACTERISTICS OF PROCEDURAL AND COGNITIVE ASSESSMENTS IN GASTROENTEROLOGY RESIDENCY TRAINING

**DOI:** 10.1093/jcag/gwad061.084

**Published:** 2024-02-14

**Authors:** J Cooper, M Gozdzik, J Silverman, K Kroeker

**Affiliations:** Adult Gastroenterology, University of Alberta Faculty of Medicine & Dentistry, Edmonton, AB, Canada; Gastroenterology, University of Alberta, Edmonton, AB, Canada; Gastroenterology, University of Alberta, Edmonton, AB, Canada; Gastroenterology, University of Alberta, Edmonton, AB, Canada

## Abstract

**Background:**

Competency based medical education has become the new standard for medical education which shifts the focus of training toward a competency, rather than time-based in framework known in Canada as ‘Competence by Design’ (CBD). CBD assesses a physician trainee’s ability to demonstrate competence in CanMEDS roles via entrustable professional activities (EPAs). EPAs utilize the O-SCORE as the metric for assessing competence. This score was developed and validated for surgical/procedural subspecialties; however, CBD currently coopts this scale for both procedural and non-procedural (cognitive) EPAs. Assessor expertise has also been shown to have an important role in performance assessments, but has not been studied in the context of CBD.

**Aims:**

Our study aims to assess for differences in O-SCORE utilization between cognitive and procedural EPAs, and whether assessor characteristics are associated with trends in assessment.

**Methods:**

Anonymized data for all Adult GI subspecialty EPAs completed from Jun 2019 to Jan 2023 at the University of Alberta was obtained. Evaluator sex, clinical vs academic practice, advanced training expertise, and EPA score was extracted. Locally a score of 5 denotes competence, while a 1-3 indicates competence was not yet achieved. A score of 4 may be accepted as evidence of competence (neutral score), at the discretion of the local competency committee. Data was analyzed via T-tests and ANOVA with post hoc Games-Howell testing with 95% confidence intervals (CI). A p-value of ampersand:003C0.05 was significant.

**Results:**

2264 EPAs were assessed including 1385 cognitive and 879 procedural EPAs. The number of EPAs completed by evaluators ranged from 11 to 165 with a mean of 60 (standard deviation: 40). Results of O-SCORE usage is summarized in Figure 1A-B. The majority of EPAs indicate competence, with 20-25% neutral, and ampersand:003C10% did not achieve competence. Less than one of third of evaluators utilized a score of 1 or 2 across all EPAs, and zero evaluators utilized a score of 1 for cognitive EPAs. Most commonly evaluators to utilized 3/5 options of the O-SCORE. Separated by EPA type, it was most common to utilize 2/5 and 4/5 options for cognitive and procedural EPAs respectively. Results of demographic comparisons are outlined if Figure 1C-E. Male and clinical evaluators submitted higher scores on average. Hepatologists submitted higher scores than all other advanced training areas for total, cognitive, and procedural EPAs.

**Conclusions:**

Across total, cognitive, and procedural EPAs there are low rates in the utilization of the whole O-SCORE scale, and our study highlights a discrepancy between procedural and cognitive EPAs. In addition, there small but significant differences in the mean EPAs score awarded between different evaluator demographics (male, clinical, hepatologists providing higher scores).

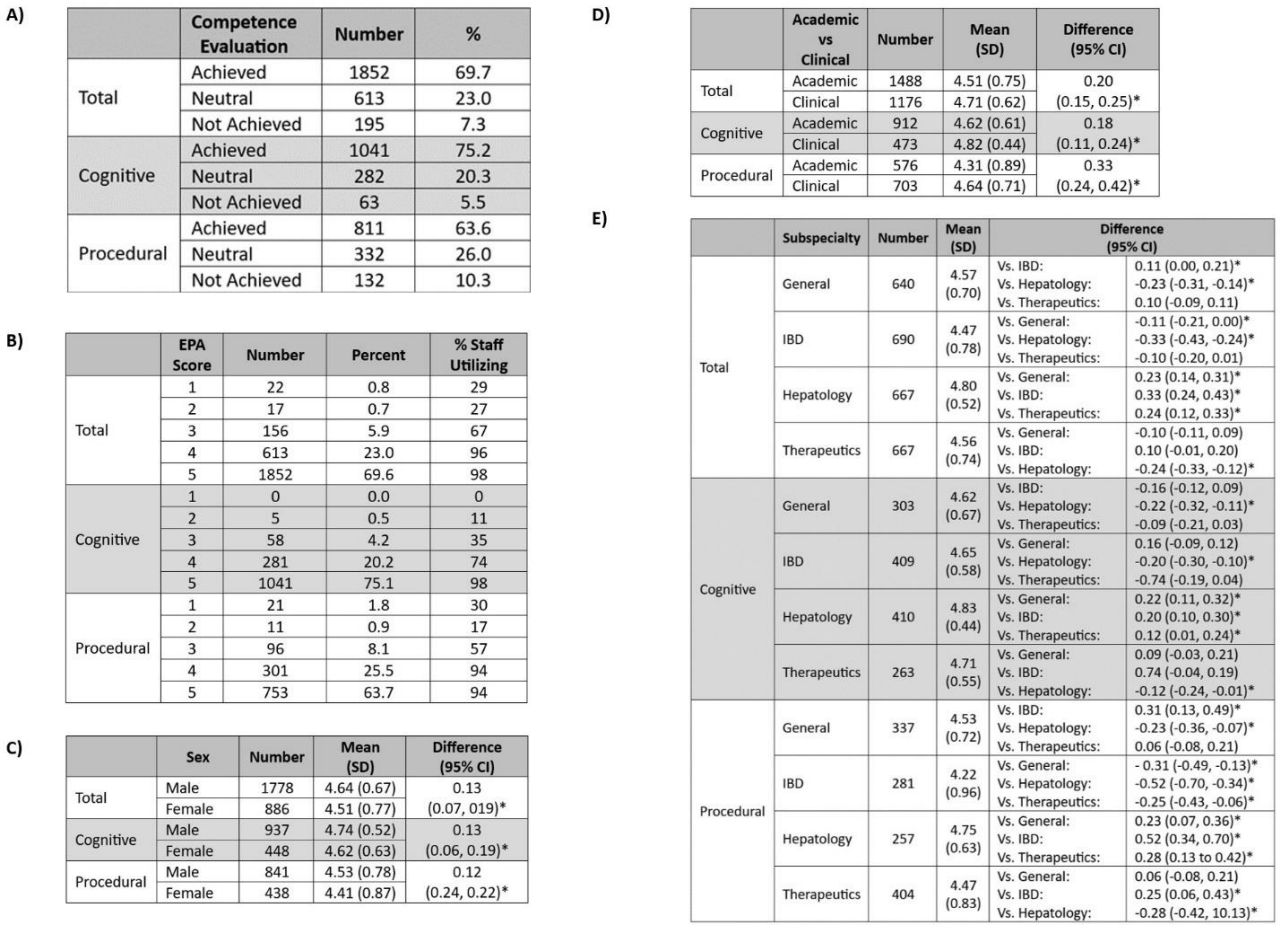

**Figure 1. A)** Number and proportion of Entrustable Professional Activities (EPA) stratified by type and competence evaluation. **B)** Number and proportion of Entrustable Professional Activities (EPA) stratified by type with scored 1-5 and percent (%) of staff utilizing each score stratified by EPA type **C)** Number, mean, and mean difference of Entrustable Professional Activities (EPA) stratified by evaluator sex and EPA type. **D)** Number, mean, and mean difference of Entrustable Professional Activities (EPA) stratified by evaluator academic vs clinical status and EPA type. **E)** Number, mean, and mean difference of Entrustable Professional Activities (EPA) stratified by evaluator advanced training and EPA type. CI: Confidence interval; SD: standard deviation; *: pampersand:003C0.05

**Funding Agencies:**

None

